# Distinct BOLD fMRI Responses of Capsaicin-Induced Thermal Sensation Reveal Pain-Related Brain Activation in Nonhuman Primates

**DOI:** 10.1371/journal.pone.0156805

**Published:** 2016-06-16

**Authors:** Abu Bakar Ali Asad, Stephanie Seah, Richard Baumgartner, Dai Feng, Andres Jensen, Elaine Manigbas, Brian Henry, Andrea Houghton, Jeffrey L. Evelhoch, Stuart W. G. Derbyshire, Chih-Liang Chin

**Affiliations:** 1 Translational Biomarkers, Merck Research Laboratories, MSD, Singapore, Singapore; 2 Biometrics Research, Biostatistics & Research Decision Sciences, Merck Research Laboratories, Merck & Co., Rahway, NJ, United States of America; 3 Early Discovery Pharmacology, Merck Research Laboratories, MSD, Singapore, Singapore; 4 Imaging, Maccine Pte Ltd, Singapore, Singapore; 5 Early Discovery Pharmacology, Merck Research Laboratories, Merck & Co., West Point, PA, United States of America; 6 Translational Biomarkers, Merck Research Laboratories, Merck & Co., West Point, PA, United States of America; 7 Dept of Psychology, National University of Singapore, Singapore, Singapore; 8 A*STAR-NUS Clinical Imaging Research Centre, Singapore, Singapore; Indiana University, UNITED STATES

## Abstract

**Background:**

Approximately 20% of the adult population suffer from chronic pain that is not adequately treated by current therapies, highlighting a great need for improved treatment options. To develop effective analgesics, experimental human and animal models of pain are critical. Topically/intra-dermally applied capsaicin induces hyperalgesia and allodynia to thermal and tactile stimuli that mimics chronic pain and is a useful translation from preclinical research to clinical investigation. Many behavioral and self-report studies of pain have exploited the use of the capsaicin pain model, but objective biomarker correlates of the capsaicin augmented nociceptive response in nonhuman primates remains to be explored.

**Methodology:**

Here we establish an aversive capsaicin-induced fMRI model using non-noxious heat stimuli in Cynomolgus monkeys (n = 8). BOLD fMRI data were collected during thermal challenge (ON:20 s/42°C; OFF:40 s/35°C, 4-cycle) at baseline and 30 min post-capsaicin (0.1 mg, topical, forearm) application. Tail withdrawal behavioral studies were also conducted in the same animals using 42°C or 48°C water bath pre- and post- capsaicin application (0.1 mg, subcutaneous, tail).

**Principal Findings:**

Group comparisons between pre- and post-capsaicin application revealed significant BOLD signal increases in brain regions associated with the ‘pain matrix’, including somatosensory, frontal, and cingulate cortices, as well as the cerebellum (paired t-test, p<0.02, n = 8), while no significant change was found after the vehicle application. The tail withdrawal behavioral study demonstrated a significant main effect of temperature and a trend towards capsaicin induced reduction of latency at both temperatures.

**Conclusions:**

These findings provide insights into the specific brain regions involved with aversive, ‘pain-like’, responses in a nonhuman primate model. Future studies may employ both behavioral and fMRI measures as translational biomarkers to gain deeper understanding of pain processing and evaluate the preclinical efficacy of novel analgesics.

## Introduction

Pain, especially chronic pain, that occurs during inflammatory or neuropathic conditions remains poorly treated. Given that approximately 20% of the adult population suffer chronic pain, there is a substantial unmet medical need for more efficacious and better tolerated therapeutics [1, 2]. In an attempt to provide better treatments, significant efforts have been devoted to establish experimental models of pain in both animals [3, 4] and humans [5–8], including nerve ligation, various neuropathy models, acute chemotherapy-induced neuropathy, and neuropathy as a result of HIV medication. Often these models of pain are combined with mechanical or thermal stimulation either to examine evoked reflex responses in animals or altered psychophysics in humans, and to characterize allodynia and/or hyperalgesia symptoms that commonly manifest in numerous clinical pain disorders including neuropathic, inflammatory and osteoarthritic pain [4, 9, 10].

During the state of allodynia, an innocuous stimulus is perceived as painful, whilst hyperalgesia results in potentiation of an already painful stimulus. Primary hyperalgesia and allodynia to heat, tonic pressure and touch occurs at the site of tissue injury as a result of peripheral sensitization of nociceptive neurons, whereas central sensitization causes secondary hyperalgesia and allodynia to mechanical/tactile stimuli that extends into uninjured tissue surrounding a nerve injury. It has been shown that both primary and secondary hyperalgesia and allodynia can be experimentally induced in healthy volunteers using the heat/capsaicin sensitization model [11], where topical/intradermal administration of capsaicin induces both types of sensitization at the site of application as well as episodic burning pain sensation around the site of application [7, 12–14]. Capsaicin activates the transient receptor potential vanilloid type 1 (TRPV1) receptor, a non-selective ligand gated cation channel expressed in Aδ and C fiber nociceptors, causing depolarization to allow transduction and conduction of signal through spinal cord to the brain [15, 16]. Activation of TRPV1 receptor by capsaicin elicits a biochemical cascade, collectively known as neurogenic inflammation, leading to the release of bioactive mediators, such as calcitonin gene related peptide, neurokinin A, nitric oxide, and prostaglandins [17]. Release of such neurotransmitters and modulators lowers the activation threshold of polymodal TRPV1 receptor that provokes both peripheral and central sensitization and mediates allodynia and hyperalgesia and thus capsaicin-induced hypersensitization has been used as an experimental pain model for hyperalgesia and allodynia observed under neuropathic conditions. In fact, using psychophysical perception or withdrawal reflex as a measure of response, several experimental pain studies validated the utility of this model in evaluation of antinociceptive effects of analgesic compounds in human subjects [18–22] as well as in nonhuman primates (NHP) [23, 24] and rodents [25]. A better understanding of the neurobiological mechanism of action in the peripheral and central nervous systems elicited by a variety of painful insults are key to prescribe appropriate pain treatments, which demands objective and quantitative measurements of pain [26].

Neuroimaging techniques provide a system-level understanding of central mechanisms related to pain processing [27–32]. For example, BOLD fMRI has been used to demonstrate nociceptive processing within the trigeminothalamocortical pathway modulated by capsaicin application [33]. Likewise, numerous functional imaging studies utilized the capsaicin sensitization model to experimentally induce hyperalgesia and allodynia and identified enhanced activity in various brain [34–39] and spinal cord regions [40, 41]. These approaches have assisted tremendously in advancing our knowledge of pain physiology and offer essential tools to evaluate experimental therapeutics with the possibility of providing congruent endpoints across species including human.

To date, however, functional neuroimaging studies of experimental pain models in animals primarily investigated acute physiologic pain from brain activation under noxious thermal and electrical stimuli [42–46], while leaving the pathophysiologic condition of chronic pain mostly unexplored. In consequence, despite these efforts providing novel information about CNS processing of nociception, application of these pain models in predicting clinical efficacy of experimental compounds have rendered limited success [3, 47, 48]. Evidently, neuroimaging studies in animal models that better reflect the underlying pathophysiology of chronic neuropathic pain and afford translatable biomarkers would be highly valuable [6, 49]. Preclinical evaluation of novel therapeutics using such animal models traditionally only examined the behavioral response and provided minimal information on the central mechanism of analgesic action. For example, tail-withdrawal latency has been commonly used as an efficacy measure in nonhuman primate and rodent models of capsaicin-induced thermal hyperalgesia and allodynia [50–54]. Additionally, the limited attempts toward neuroimaging characterization of central capsaicin response only included rodents and focused either on the C-nociceptor pain caused by capsaicin administration [55] or aspects of secondary hyperalgesia [56, 57]. Despite the attractiveness of nonhuman primates in neuroscience research arising from the similarity in cytoarchitecture and functional connections with human brain, present literature lack neuroimaging evidence of capsaicin-induced sensitization in nonhuman primates.

In this study, we sought to provide the first neuroimaging evaluation of the capsaicin heat fMRI model in nonhuman primates, with tail-withdrawal measurements conducted on the same animals. We hypothesized that in nonhuman primates, topical capsaicin administration should potentiate non-noxious heat-induced cortical activation in the brain regions previously observed in human pain fMRI experiments. In conjunction with behavioural assays (e.g., tail-withdrawal), this imaging model could offer a translational tool to bridge the gap between preclinical research and clinical investigation and provide better understanding of the mechanisms of novel treatments for pain.

## Material and Methods

### Ethics Statement

The current study, in addition to its protocol, was approved by the Institutional Animal Care and Use Committee (IACUC) at Merck & Co (Permit Number: 11107413710013). Testing facilities at Maccine (Maccine Pte Ltd, Singapore) were accredited by the Association for the Assessment and Accreditation of Laboratory Animal Care (AAALAC) and this study was approved by the IACUC at Maccine (protocol number: 247–2011). No animals were sacrificed for the purpose of this experiment. Trained veterinarians and animal technicians were involved in the care of the animals and in the oversight of all animal procedures performed.

### Animal

Adult female Vietnamese Cynomolgus monkeys (*Macaca fascicularis*; n = 8, age = 6–7 years, body weight = 3–4 kg) were used for this study. Animals were pair housed in temperature- (18–26°C) and humidity- (30–70%) controlled rooms maintained on a 12:12 light/dark cycle, with lights on at 7:00AM. Monkey chow free of animal protein was offered twice daily, as well as a controlled amount of fruits or vegetables. Aside from daily fruit rations, frozen homemade treats (i.e., fruits, raisins, cereals, etc.) were provided once a week. Mains tap water was offered *ad libitum*. Prior to the commencement of the imaging study, a complete physical examination, including hematological/blood chemistry analysis, was performed and reviewed by the attending veterinarian. All animals were acclimatized to the animal facility for a minimum period of two weeks before the beginning of experimental procedures. The animals were provided with cage toys (e.g., mirrors, kong toys, flexi keys) in addition to the use of radios and televisions for environmental enrichment and welfare and psychological well-being was ensured by having daily cage side observation of the animals. To avoid any potential adverse events such as thermal burn or irritation from capsaicin application, the site applied with thermal challenge was examined immediately after the scan, then 2–3 hours after the scan. After recovery from anesthesia the animals were checked for general condition at least two times within the following 12 hours. During the study, there was no report of any abnormality, no tissue damage created by the thermal testing, short action span of capsaicin and no distress or pain being observed in all animals when examined by the veterinarian. Finally, since there were no clinical signs in the animals that indicated the animals experiencing pain, no analgesics were administered in these animals.

### Thermal Stimulation

The thermal stimulation in fMRI experiment was delivered to the palmar surface of the proximal half right forearm using a computer-controlled MRI-compatible peltier element based, 1.6×1.6 cm^2^, heat probe (ATS Thermode) (Pathway, Medoc, Ramat Yishai, Israel) whereas the thermal stimulation in tail withdrawal assay was delivered using a heated water bath. The Pathway system is capable of delivering thermal stimuli via ATS thermode at temperature ranging between 0°C– 55°C at slew rates up to 8°C/s, which can be triggered by the MRI scanner. It was found previously that nociceptive fibers in monkeys show little or no response to stimulus temperatures less than 45°C [58, 59]. Correspondingly, in humans, non-noxious warm stimuli were induced at temperatures between 40~43°C, whilst noxious pain stimuli were produced at higher temperatures (i.e., 46~50°C) [60–64]. Based on these previous findings, we selected 42°C as the non-noxious and 48°C as the noxious heat stimulus for the tail withdrawal experiment. For the fMRI study, we selected 42°C temperature both before and after topical capsaicin application since the animal could not withdraw the stimulated limb under anesthesia. Following the topical capsaicin application it was assumed that 42°C heat stimulus would produce noxious pain sensation as reported in human studies [33].

### Capsaicin Formulation

Formulation of capsaicin (8-Methyl-N-vanillyl-trans-6-nonenamide; Sigma-Aldrich, St. Louis, MO) solution for subcutaneous injection at the tail during tail withdrawal study was based on an established capsaicin formulation protocol [51, 65, 66] whereby 1 mg/mL capsaicin was dissolved in a solution of Tween 80/ethanol/saline (volumetric ratio: 10%/10%/80%) giving 0.1% w/v solution. On the other hand, capsaicin was topically administered as a patch during fMRI. The solution for patch was prepared by dissolving 1 mg/mL capsaicin in 70% ethanol excipient (0.1% w/v solution) as described by Mohr et al., [67] in a previous human fMRI study. For both route of administration, 0.1 mL capsaicin solution was used as a dosing volume. Of note, all the excipient and diluent used during the preparation of capsaicin solution was pharmaceutical grade (USP/BP) or analytical research grade. In addition, capsaicin was formulated in a single step compounding under sterile environment.

### Tail Withdrawal Behavioral Study

Prior to assessing responses to the heat stimuli, animals were habituated to the chair restraining and experimental procedures according to previously described methods [68]. Briefly, animals were first acclimatized to the water bath via immersion for a maximum of 20 s prior to the baseline and capsaicin trials. In the baseline study, for each animal the distal 1/3 portion of the shaved tail (~15 cm from the tip) was immersed into one of the two water baths heated to either 42°C or 48°C. The order of immersion was randomized and three measurements per animal were recorded at both temperatures. Animals that failed to withdraw within 20 s had their tails manually removed from the water bath and a latency score of 20 s recorded. On a separate testing day, all procedures were repeated 15 min after a subcutaneous injection of 0.1 mg capsaicin, in a dosing volume of 0.1 mL, into the distal 1/3 portion of the tail and the tail was immersed for a maximum of 20 s according to the same procedure.

### Capsaicin Heat Challenge fMRI Study

#### Animal Preparation

Each animal was fasted for a minimum of six hours and then injected with ketamine (10 mg/kg IM) to induce anesthesia. Once anesthetized, animals were subsequently placed on the preparation table for the insertion of endotracheal tube and the proximal half of the right forearm was shaved in order to apply the thermal challenge. Ear plugs were placed in each ear canal and eye ointment applied. Animals were then transferred and placed on the scanner bed in a head-first supine position. The animal’s head was immobilized to minimize motion artifacts. During the MRI scan, animals were mechanically ventilated to maintain 20 ± 1 stroke/min using medical air (1.3~1.5 L/min) by an MRI-compatible ventilator (SurgiVet, Dublin, OH) and vital signs were monitored by an MRI-compatible physiological monitoring system (Datex-Ohmeda, GE Healthcare, WI). Anesthesia was maintained through the inhalation of ~1% isoflurane (inspired and end-expired concentration of isoflurane was 1.1 ± 0.01% and 0.9 ± 0.01% (mean ± SD), respectively). Additionally, animals were covered using a warm air heated blanket (ThermaCare Convective Warming System, Gaymar Industries, NY) to maintain rectal temperature between 36.5~37.5°C (mean ± SD = 37.1 ± 0.3°C) during the imaging experiment (measured using fiber-optic temperature sensor system manufactured by Pico-M, OpSens, Quebec, Canada).

Heart rate and SpO_2_ appeared to be stable and within normal ranges [69–72] throughout the imaging period (mean ± SD, heart rate = 161 ± 12 and SpO_2_ > 95% (98.7 ± 1.3)). The level of end-tidal CO_2_ (EtCO_2_) was continuously measured from airway by sampling the gas via a side-stream port located at the endotracheal tube/breathing circuit interface. The EtCO_2_ level corresponded to 35 mmHg– 45 mmHg partial pressure of CO_2_ in arterial blood (PaCO_2_) which was established in a separate arterial blood gas sampling experiment (data not shown).

#### Thermal Challenge Paradigm and Capsaicin Application

**Fig 1:**
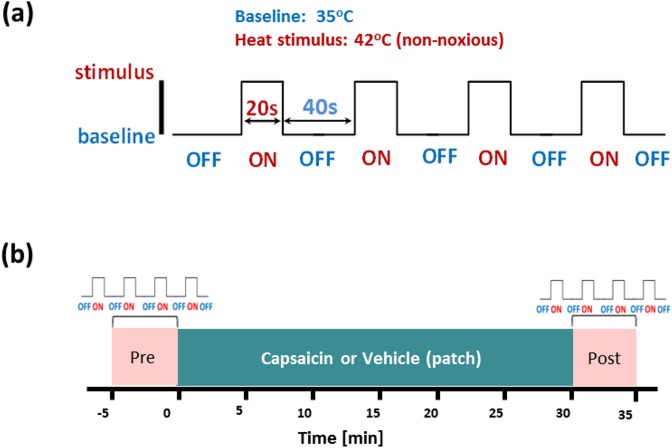
Capsaicin pain fMRI experimental protocol with heat challenge. (a) The heat stimulation paradigm used in the imaging study. The baseline (OFF) temperature was 35°C (40 s), while heat stimulus (ON) at 42°C (20 s) were exerted using a thermode placed at the left forearm of the animal. Four cycles were repeated for each individual trial. (b) The imaging protocol used in the capsaicin-heat BOLD fMRI experiment. Imaging data were acquired during the baseline (‘Pre’) and post -capsaicin or -vehicle patch application (‘Post’). The capsaicin solution was delivered via a patch (0.1 mg of capsaicin in a dosing volume of 0.1 mL) attached at the location where the thermode was placed.

The ATS thermode was secured with a Velcro strap at the palmar surface of the proximal half of the right forearm. During the imaging study, the thermode maintained a steady baseline temperature of 35°C for 40 s (OFF) during rest and a steady temperature of 42°C for 20 s (ON) during stimulation periods. Each run included 4 cycles as shown in Fig 1A. All animals completed one run and were then randomized to receive either vehicle or capsaicin solution before completion of a second run (Fig 1B). Consistent with previous methods [73], capsaicin or vehicle solution (0.1% w/v, 0.1 mL) was dripped on a gauze patch (1.5×1.5 cm^2^ cutout), with a homogeneous spread. The patch was then attached to the site of thermal challenge of the right forearm, and covered by a latex-tape to minimize evaporation, for approximately 30 min. After the removal of the tape and patch, the thermode was re-attached to the right forearm and the second run was recorded for the post-capsaicin thermal challenge.

#### BOLD fMRI Data Acquisition

All imaging experiments were conducted on a 3 Tesla MRI scanner (Siemens Medical Solutions, Erlangen, Germany) using a dedicated 8-channel phased-array head coil (RAPID Biomedical GmbH, Germany). High resolution T_1_–weighted anatomical images with good tissue contrast were acquired using a 3D MPRAGE sequence (TR/TE = 2 s / 5.7 ms, in-plane resolution = 0.33 × 0.33 mm^2^ (with interpolation), slice thickness = 2.2 mm and 24 slices). BOLD fMRI data was collected using single-shot gradient-echo EPI pulse sequences (TR/TE = 3 s /30 ms, in-plane pixel size = 1×1 mm^2^, slice thickness = 2 mm, and 24 slices with 0.2 mm slice gap). The fMRI protocol for capsaicin-induced hypersensitization study included (*i*) BOLD data acquisition with 20 s 42°C heat stimulus, interleaved with 40 s 35°C (‘Pre’), at the forearm (see Fig 1A) (*ii*) topical capsaicin or vehicle patch application (30 min) on the same site of thermal stimulation, and (*iii*) repeat BOLD data acquisition with 20 s 42°C heat stimulus, interleaved with 40 s 35°C (‘Post’). Ninety-nine imaging volumes were acquired pre- (run 1) and post-capsaicin or vehicle (run 2). Due to technical constraints in the availability of animal cohort and imaging scanner, post-vehicle data were only recorded from five of the eight animals.

#### Data Analysis

Data analyses were conducted using FMRIB’s Software Library (FSL) (http://www.fmrib.ox.au.ul/fls) [74] and in-house Matlab (MathWorks, Natick, MA) programs. Functional brain images obtained from every animal were first preprocessed by extracting the skull and other non-brain regions using FMRIB’s brain extraction tool (BET, [74]), while motion correction was performed using FMRIB’s Linear Image Registration Tool (MCFLIRT, [75]) on each EPI volume. In addition, a spike detection algorithm was used to identify abrupt head motion. All imaging volumes were then spatially smoothed with a 2-mm full-width-half-maximum (FWHM) Gaussian spatial filter, and the time series at each voxel temporally filtered using a 0.01 Hz high-pass filter to remove low-frequency temporal noise and drift of fMRI data. Following these preprocessing steps, subject-level GLM analysis of fMRI data was carried out with FSL’s fMRI Expert Analysis Tool using FMRIB’s Improved Linear Model (FEAT FILM, [76]) with nonparametric estimation of time series autocorrelation to locally pre-whiten voxel-wise time series. To perform unbiased univariate linear regression analysis, nuisance regressors were prepared to exclude confounding explanatory variables (EVs) using six motion parameters derived from head movement (three translations and three rotations). Mean time-course signals extracted from ventricles and white matter were also included to regress out additional confounds related to physiological noises and the linear drift.

For the group-level analyses, functional data of each animal were first co-registered to individual’s anatomical images with a 6 degree-of-freedom rigid body transformation. Subsequently, co-registration to a template monkey brain in MNI space (a standard created by Montreal Neurological Institute (MNI), [77]) was performed using FMRIB’s Linear Image Registration Tool (FLIRT, [75]) with a 12 degree-of-freedom affine transformation. The calculated transformation matrix parameters were then applied to the functional dataset to perform group-level analyses in MNI standard space. The absence of vehicle data for three of the animals meant that a fully factorial repeated measures analysis could not be completed. Consequently, group comparisons were conducted using a mixed-effects (FSL FLAME 1+2) paired *t*-test to determine the group mean of the differential BOLD responses to the 42°C stimulus pre- and post-capsaicin and pre- and post-vehicle separately (see supplementary material for a full factorial analysis from the five animals completing all procedures). The final group activation maps were thresholded at *z > 2*.*3* with differences considered statistically significant at *p < 0*.*02* [63], in order to identify brain regions showing potentiated heat BOLD responses under capsaicin-induced hypersensitization.

In addition, region-of-interest (ROI) analyses were performed using a standard monkey brain atlas [77]. Regional percentage BOLD signal changes were extracted using the same atlas from selected anatomical brain regions under each condition (42°C vs 35°C heat stimulus before and post-capsaicin or vehicle). The regions selected were based on previous publications [32, 59, 78–82], including vermis, cerebellum, anterior cingulate cortex, posterior cingulate cortex, insula, thalamus, primary and secondary somatosensory cortices and frontal cortex. The effects of capsaicin (versus vehicle) and regions were determined using repeated measures ANOVA implemented in SPSS (Statistics for Research & Analysis V21).

## Results

### Tail Withdrawal Test

**Fig 2:**
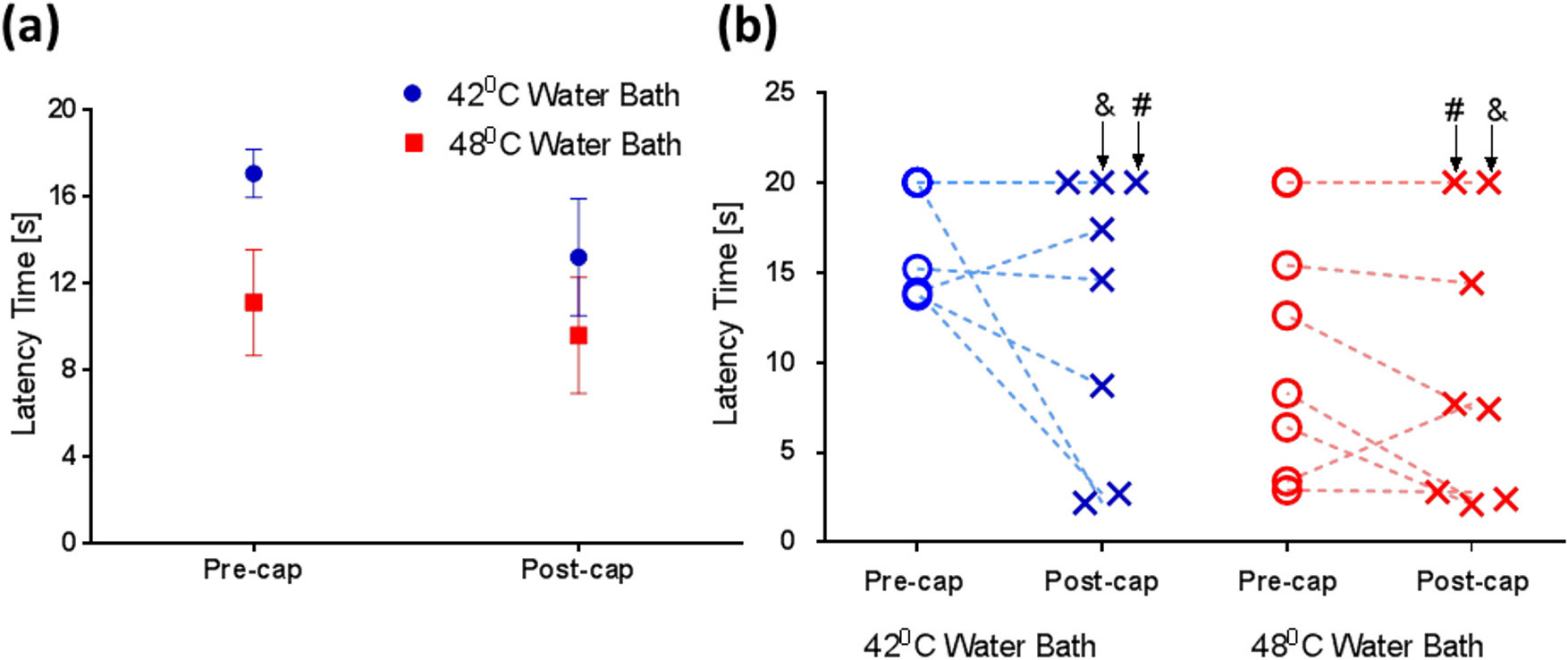
The effect of temperature and capsaicin on tail withdrawal latency. (a) Our data show that compared to the baseline (‘Pre’), decreases in latency time (mean ± SEM, n = 8) were found after capsaicin application (‘Post’) at both temperatures and the effect of temperature was significant (F1,7 = 7.89, p = 0.026); however, there was no effect of capsaicin (F1,7 = 2.69, p = 0.145). (b) Tail withdrawal latency time measured from individual animals highlight the inter-subject variability, with two ‘non-responders’ (labelled by ‘#’ and ‘&’) identified (no response to the thermal challenge at both temperatures).

Fig 2A shows the average tail withdrawal latencies (mean ± SEM at 42 / 48°C water baths) at baseline (17.09 ± 1.11 s / 11.12 ± 2.45 s) and 30-min post (13.21 ± 2.70 s / 9.59 ± 2.68 s) the subcutaneous injection of capsaicin. Our results indicate that capsaicin reduced the latency at both temperatures, although formal analysis failed to demonstrate significance for the main effect of capsaicin (F_1,7_ = 2.69, *p* = 0.145). There was a significant main effect of temperature (F_1,7_ = 7.89, *p* = 0.026) but no significant interaction of capsaicin with temperature (F_1,7_ = 1.15, *p* = 0.319). Fig 2B shows the tail withdrawal latencies pre- and post-capsaicin administration from individual animals. Interestingly, it is noted that two animals that did not respond to the thermal challenge (both 42 and 48°C water baths) at baseline and also failed to react to the potentiation effect of capsaicin.

### fMRI Analysis

**Fig 3:**
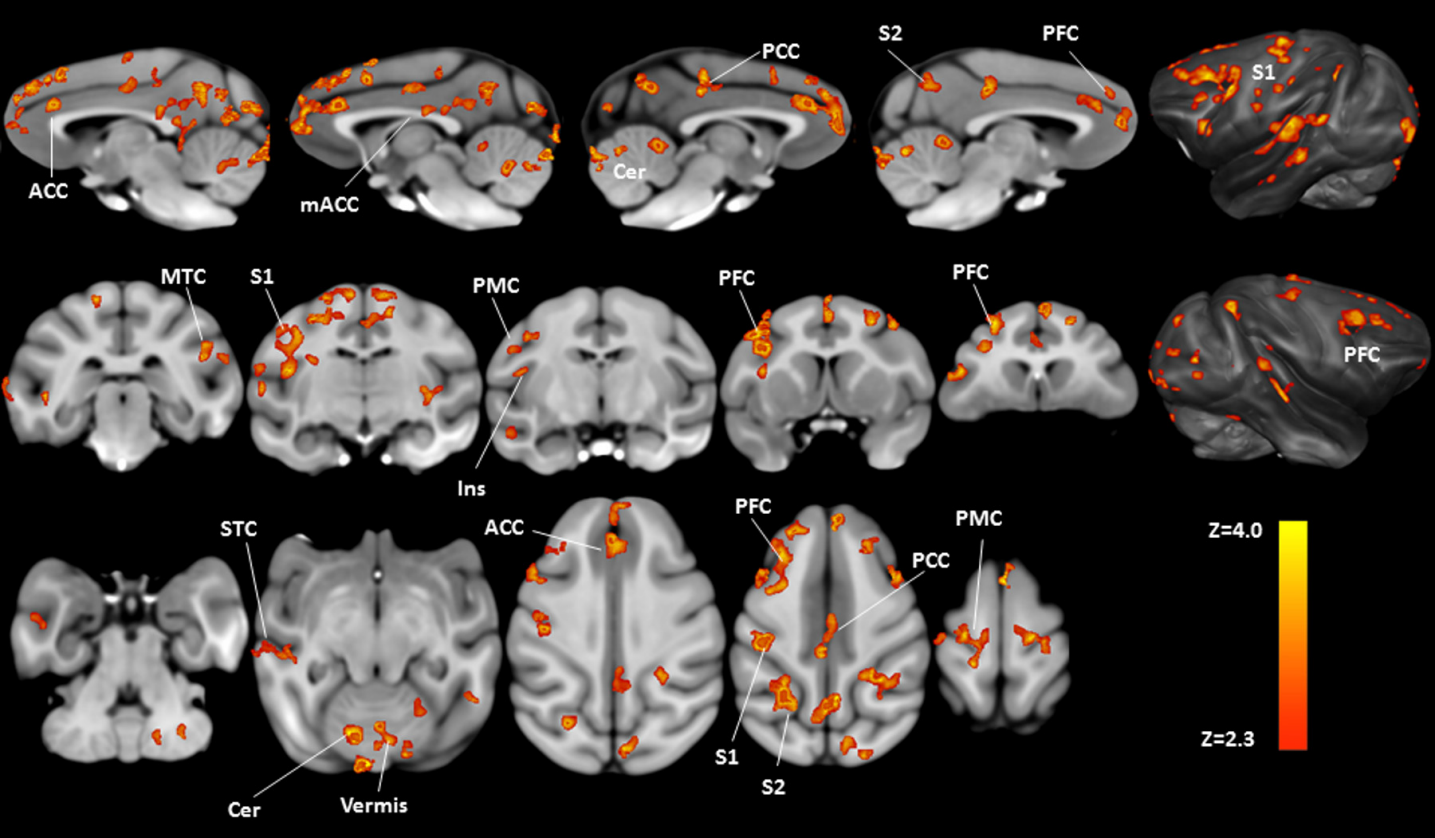
Group comparisons of brain activation patterns showing the effect of capsaicin-induced hypersensitization on heat fMRI signals. After the topical application of capsaicin, significant increases in BOLD response to 42°C challenge can be found in several pain-related brain regions (pre- versus post-capsaicin application, paired t-test, p<0.02, n = 8). Labeled brain areas in neurological orientation are: ACC = anterior cingulate cortex; mACC = mid anterior cingulate cortex; PCC = posterior cingulate cortex; S2 = secondary somatosensory cortex; PFC = prefrontal cortex; S1 = primary somatosensory cortex; MTC = mid temporal cortex; Ins = insula; PMC = primary motor cortex; STC = superior temporal cortex; Cer = cerebellum.

BOLD fMRI responses were first investigated on a voxel-by-voxel basis using FSL and revealed significant differences when delivering 42°C heat stimulus with topical capsaicin compared to before capsaicin administration, and these differences are illustrated in Fig 3 (paired t-test, p<0.02, n = 8), showing significant potentiated BOLD responses in the anterior cingulate cortex (extending into medial frontal areas), the mid-anterior cingulate cortex (extending into medial motor areas and posterior cingulate cortex), primary and secondary somatosensory cortices, insula, prefrontal cortex, and cerebellum. All responses were primarily bilateral; whilst larger activations were observed in most contralateral brain regions (the details are tabulated in Table 1). In contrast, comparisons of BOLD response during 42°C heat stimulus with vehicle application revealed no brain areas with significant potentiation (n = 5).

**Table 1:** Potentiated BOLD responses to 42°C challenge with the topical application of capsaicin (pre- vs post- capsaicin).

Region	Side	MNI coordinates (X,Y,Z) [mm]	Mean Z-score	Volume [mm^3^]
Vermis		33, 13, 20	2.8	173
Cerebellum	R	41, 14, 15	2.7	142
	L	21, 15, 13	2.8	140
Anterior Cingulate Cortex (ACC)	R	31, 70, 29	2.8	52
	L	30, 69, 29	2.9	107
Posterior Cingulate Cortex (PCC)	R	31, 45, 37	2.7	38
	L	29, 37, 37	2.7	34
Insula	L	14, 43, 26	2.5	2
Primary somatosensory cortex (S1)	R	38, 36, 42	2.7	11
	L	16, 39, 37	2.8	141
Secondary somatosensory cortex (S2)	R	41, 26, 42	2.8	290
	L	20, 28, 39	2.7	204
Primary Motor Cortex	R	36, 42, 41	2.7	117
	L	26, 42, 45	2.8	315
Superior Frontal Cortex	R	31, 60, 40	2.8	184
	L	31, 53, 42	2.8	216
Mid Frontal Cortex	R	42, 59, 35	2.6	18
	L	19, 57, 33	2.7	87
Inferior Frontal Cortex	R	36, 69, 28	2.8	8
	L	17, 54, 33	2.8	73
Superior Temporal Cortex	R	49, 39, 21	2.8	146
	L	7, 39, 28	2.7	125
Mid Temporal Cortex	R	48, 30, 29	2.8	16
	L	5, 36, 21	2.8	87
Midbrain		38, 42, 15	2.6	4

Within each brain region, the MNI coordinates (Montreal Neurological Institute standard) correspond to the location of the voxel having maximum Z-score, while the mean Z-score is computed over the activated volume.

**Fig 4:**
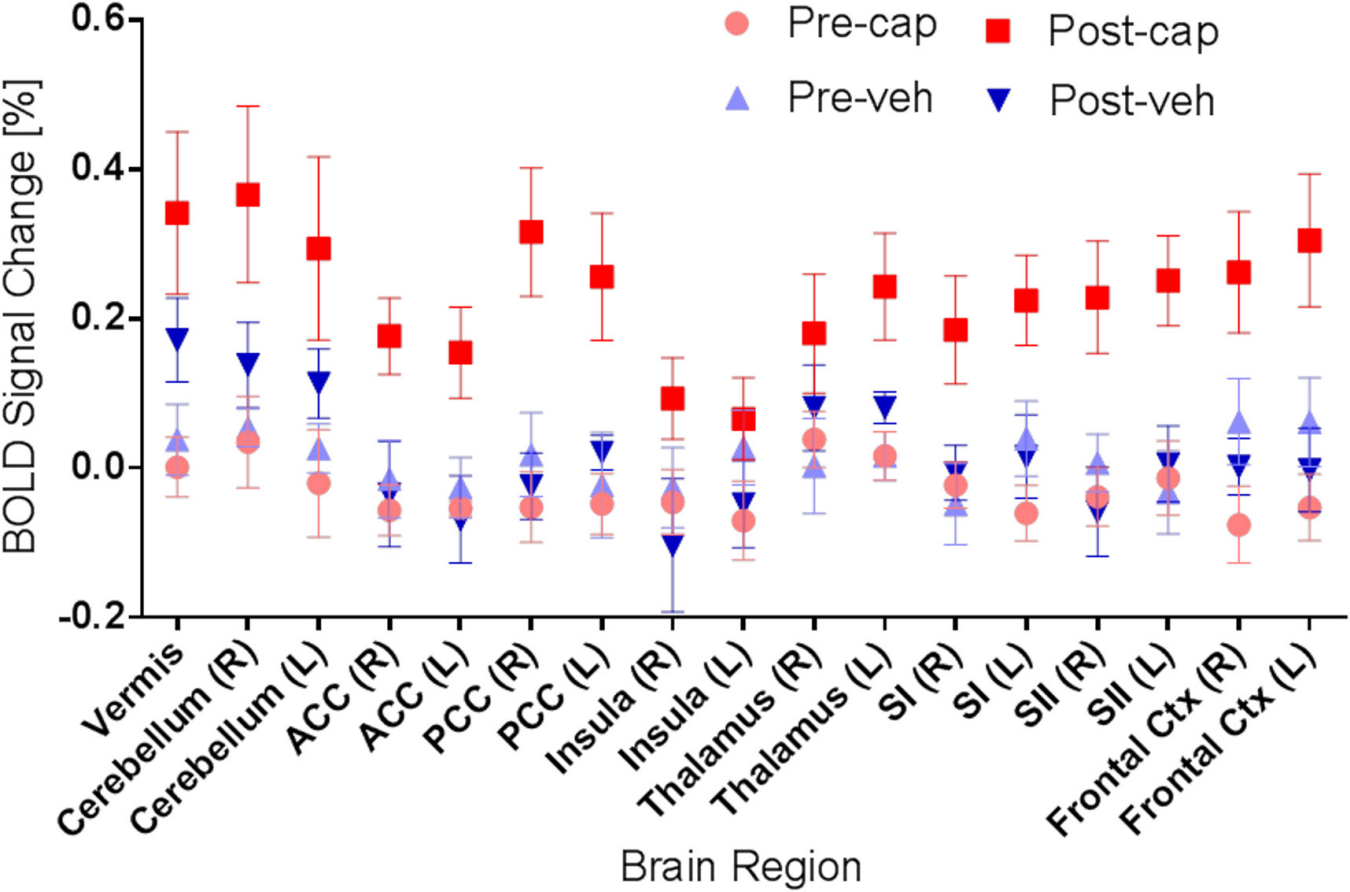
Regional BOLD signal changes induced by 42°C heat challenge at pre- and post-capsaicin application. Region-of-interest (ROI) data analyses revealed that significant increases in BOLD signal change (mean ± SEM) was found after the capsaicin (cap) application, but not vehicle (veh) treatment. Specific brain regions associated with pain perception are highlighted.

**Fig 5:**
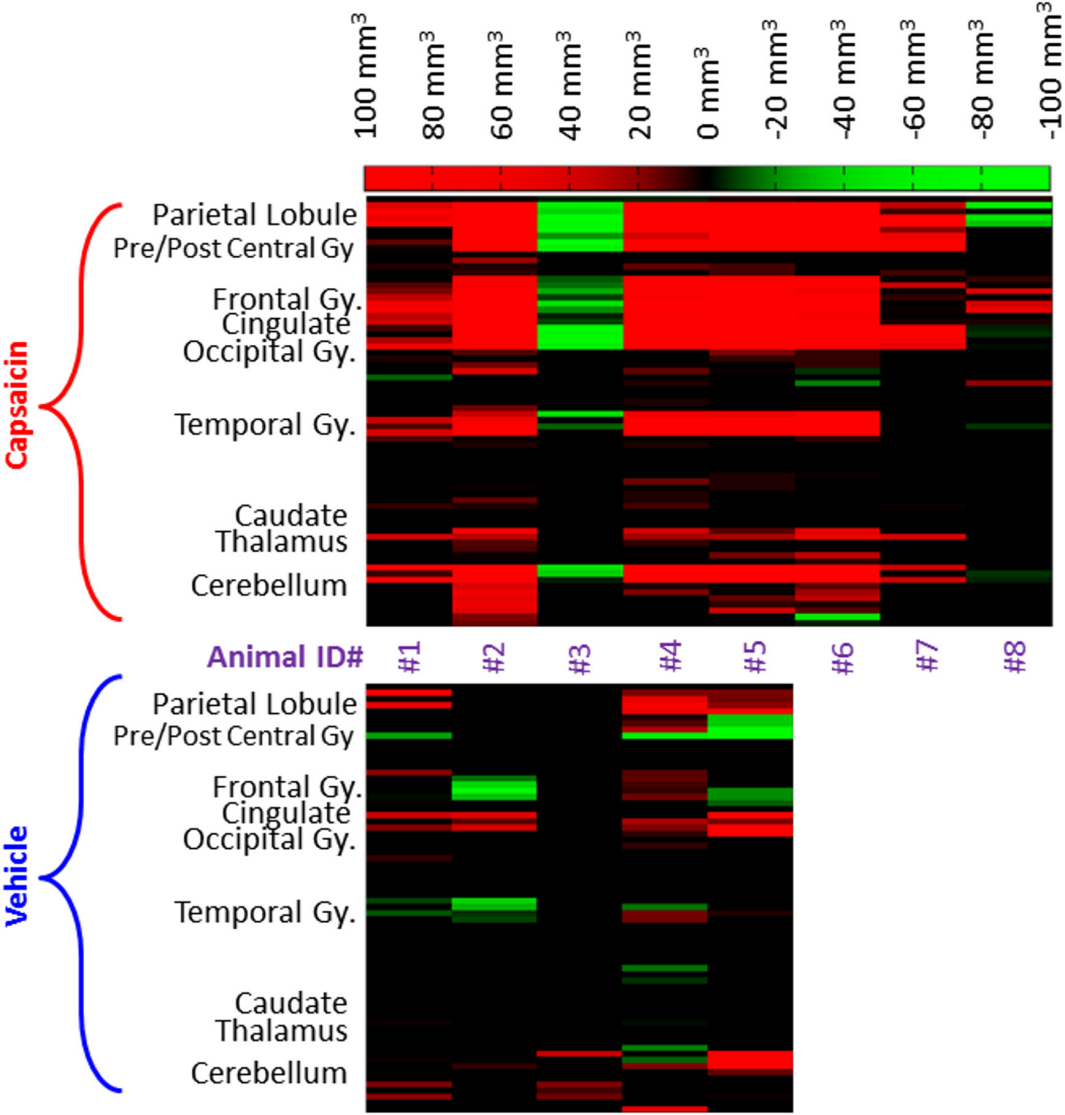
Inter-subject difference in activated brain volume potentiated by the application of capsaicin or vehicle. Differences in activated brain volume measured from individual animals, elucidating the inter-subject variability of the capsaicin effect, where red represents an increase in activated brain volume after the capsaicin application and green represents the opposite. As shown, increases in activated brain volume after the capsaicin application can be found in most animals (6 out of 8) whilst the vehicle application did not elicit robust alterations for most animals.

Regional differences of pain-related brain areas between pre- and post-capsaicin or vehicle treatment are shown in Fig 4. Percentage signal change due to the delivery of 42°C heat stimulus was extracted for each condition (pre- and post- capsaicin and pre- and post- vehicle) using the anatomical ROI and significant increases in percentage signal change following capsaicin administration can be observed in a region-specific manner. Formal analysis confirmed a significant main effect of capsaicin (F_3,12_ = 5.3, p = 0.01) and region-specificity (F_16,64_ = 5.1, p < 0.001), while the interaction of region-specificity with condition was not significant (F_48,192_ = 1.2, p > 0.2). In addition, to examine the inter-subject variability of capsaicin effect observed in the imaging experiments, differences in activated brain volume (mm^3^, post- minus pre-capsaicin) at specific brain regions were calculated from individual animals (see Fig 5), in which red represents an increase in activated brain volume after the capsaicin application and green represents decrease in activation volume.

**Fig 6:**
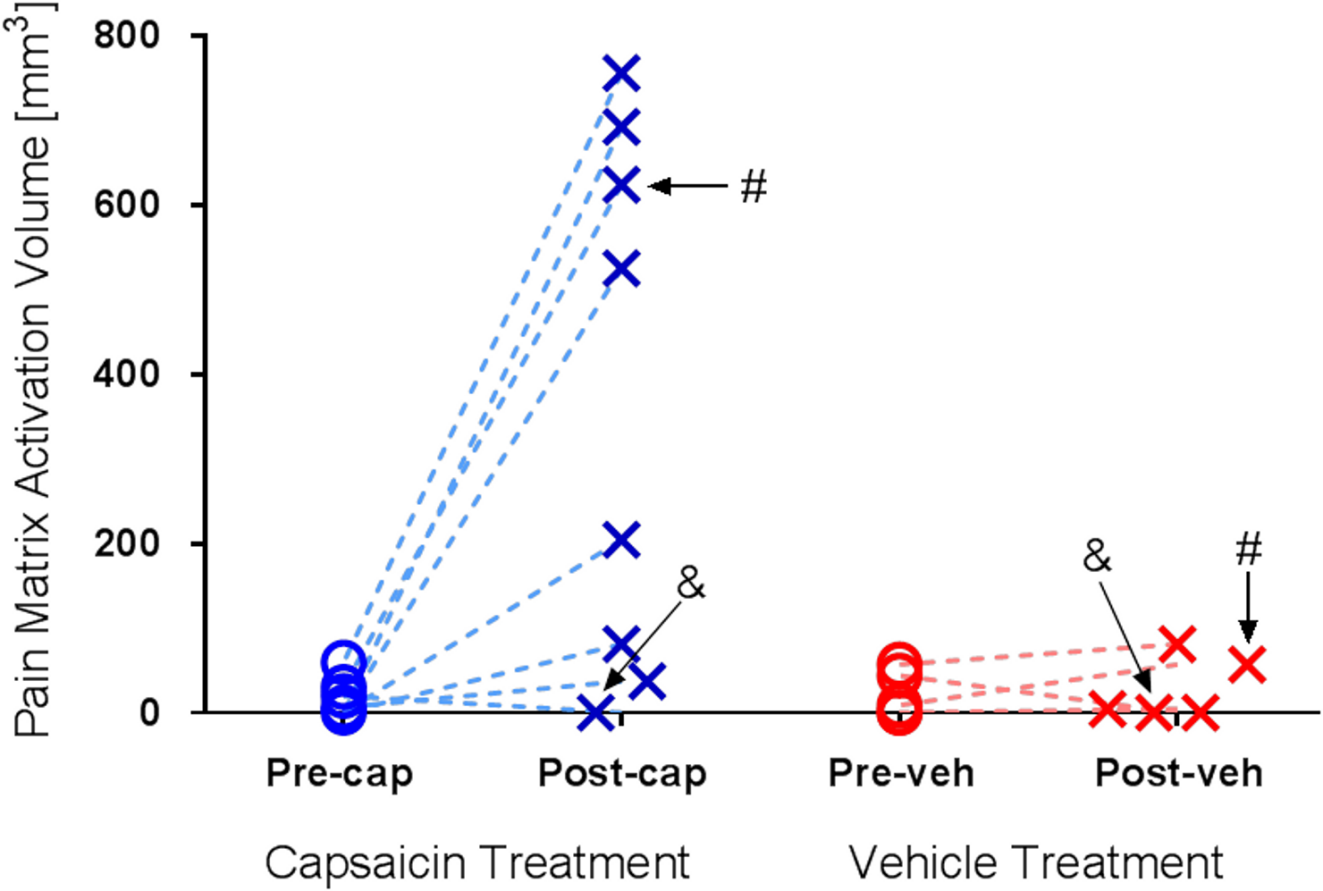
Activated brain volume within the ‘pain matrix’ observed in individual animals. 42°C heat challenge elicited minimal activation within the ‘pain matrix’ in both pre and post -capsaicin (Pre-cap and Post-cap) or -vehicle (Pre-veh and Post-veh). Variation in activated brain volumes under post-capsaicin heat challenge was observed: 4 out of 8 animals showed large increases; another 2 animals showed modest increases, and the remaining 2 animals failed to response to capsaicin challenge. Interestingly, two non-responders identified in the tail withdrawal behavioral assay (‘#’ and ‘&’, see Fig 2) showed differential responses in the imaging study.

Total activated ‘pain matrix’ volume observed in individual animals are shown in Fig 6. Interestingly, we noticed that one animal (#3) that did not respond to capsaicin application in behavioral measurements (i.e., no decrease in tail withdrawal latency time; see Fig 2B) consistently showed no effect in capsaicin-induced brain activation in the imaging experiments (see Figs 5 and 6). However, the other non-responder identified in the tail withdrawal study (#4) showed significant and robust increases in BOLD signal change after the capsaicin application. For the vehicle application, no significant change in activated brain volume was found among most brain regions. This finding is consistent with the data shown in Figs 5 and 6, in which only 4 out 8 animals showed large increases in activated brain volume after the capsaicin application and another 2 animals showed modest increases.

## Discussion

The main finding of this NHP imaging study is increased BOLD responses to 42°C heat stimulus following capsaicin application in cortical regions that are known to associate with pain experience in humans [32, 59, 78, 82]. Specifically, following the topical application of capsaicin, significantly greater activation of pain-related brain regions (the ‘pain matrix’) during delivery of a 42°C heat stimulus to the right forearm was found. Significantly activated regions included the prefrontal cortex, anterior and posterior cingulate cortices, primary and secondary somatosensory cortices, insula and cerebellum. It is noteworthy that these regions were not significantly responsive to 42°C heat stimulus without capsaicin application, and administration of vehicle also did not result in significant potentiation of BOLD responses. Results of ROI analysis confirmed the main effect of capsaicin (see Table 1, Fig 4). Direct full factorial comparison of capsaicin and vehicle effects on a voxel-by-voxel basis are provided for the five animals completing the entire study and confirm the same findings (see S1 and S2 Figs).

Previously, several imaging studies in humans have demonstrated activation of primary and secondary somatosensory cortices, anterior cingulate cortex, insula and prefrontal cortex during pain experience (for reviews, see Peyron et al. [81]). For example, in the study reported by Becerra et al. [83], large activation in pain-related brain areas was observed following a painful thermal stimuli delivered at 46°C. In contrast, application of non-painful 41°C thermal stimuli failed to produce such results, indicating a distinct difference in activation map between painful vs non-painful stimuli. The heat stimuli used in the current study was 42°C, which is generally perceived as hot to mildly painful in humans [63] but is below the range of temperatures typically selected as painful heat in human pain fMRI studies [84, 85]. Despite the use of a hot to mildly painful stimuli, the presence of capsaicin potentiated the response and revealed an overlapping activation in a NHP model to painful stimuli in humans. Therefore, the observed brain activation pattern that resembles painful stimuli likely represents the pain caused by heat stimuli applied to the neural tissue undergoing capsaicin-induced peripheral sensitization. We anticipated that the use of capsaicin in this NHP model would cause peripheral sensitization leading to allodynia and hyperalgesia, which are common symptomatic manifestations of neuropathic pain. Previous studies using capsaicin-induced hyperalgesia in healthy volunteers have demonstrated increased brain activation predominantly in the anterior, or perigenual, regions of the cingulate cortex and the dorsolateral prefrontal cortex [86], similar to the anterior cingulate cortex (ACC) and prefrontal cortex (PFC) activations reported here (Fig 3). In addition, resonating with many earlier reports, we have also observed robust activation in primary, secondary and pre- motor cortical areas within superior frontal, medial frontal and precentral gyri [79, 87–90] and superior and middle temporal gyri [32, 83] (see Table 1). Some of these brain regions are implicated in the descending control of pain perception [91, 92], while the widespread activation across various regions within frontal cortices and cerebellum have been identified as another signature of clinical chronic pain conditions [33, 39, 93].

This study provides the first evidence that capsaicin potentiated nociceptive processing in NHP elucidates similar BOLD activation to those seen in humans and thus enables the possibility for modeling mechanisms associated with human pain experience in nonhuman primates. This is a step forward from the earlier demonstration of neuroimaging studies in animal models of pain, where, firstly, it expands beyond the commonly used thermal pain stimuli by including the capsaicin-induced hypersensitization to mimic symptoms observed in neuropathic pain and secondly, instead of rodents, neuroimaging characterization of central capsaicin response in NHPs was performed. Another interesting aspect of this work is the demonstration of primary hyperalgesia and allodynia, whereas the earlier rodent studies mainly included nociceptive pain or aspects of secondary hyperalgesia and allodynia following the administration of capsaicin. It is noted that substantial differences in brain activation pattern between primary and secondary hyperalgesia were found in human [35], highlighting the need to study both types of hyperalgesia for improving our understanding of neuropathic pain mechanisms.

Delivery of 42°C relative to 35°C heat stimulus did not result in any significant BOLD response except after the topical application of capsaicin, while the ROI analysis (see Fig 4) did not provide evidence of even small responses to the 42°C heat stimulus, especially in S1 and S2, in the absence of capsaicin. These observations may be considered somewhat surprising as 42°C is expected to activate thermal receptors and may also activate nociceptors [94, 95] that might be expected to result in thalamus, S1 and S2 activity [35, 59, 96]. The lack of BOLD responses to 42°C heat stimulus might be due to the relatively limited number of animals used in our study or due to the use of anesthesia to immobilize the animals during all fMRI procedures. Sedation is known to suppress cognitive, affective and other responses to stimulation, which appears to reduce BOLD responses [97].

Level of anesthesia is indeed critical to observe fMRI responses, while both types of anesthesia and selected doses need to be optimized for the specific stimuli or pharmacological challenge. In primates, it has been shown that minimum alveolar concentration (MAC) of isoflurane required to prevent any movement in response to surgical pain is 1.28% [71], which is higher than the MAC of 0.9% delivered in this study. Hence, the isoflurane dose used in our study can be considered reasonably low, however, sufficient for the maintenance and prevention of any motion from painful thermal stimulus. Various other anesthesia paradigms have been used in animal fMRI studies, e.g., combined anesthesia consisting of 0.3% - 0.5% isoflurane and various opioids [69], neuromuscular blocking agents [98], sedatives [99] and hypnotic agents [100]. However, many of these anesthetics also have analgesic effect, which may impart undesired pharmacological actions for applying this animal model to develop novel pain treatments.

One interesting observation from the tail withdrawal study is the presence of two non-responders (#3 and #4) that failed to show any response to both the 42°C and 48°C water baths and to the capsaicin (Fig 2B). Typically, temperatures between 46 and 50°C are considered as highly painful in healthy human volunteers [60–64] and a response from such a noxious stimuli is commonly expected. On the other hand, many tail withdrawal studies have been conducted in water baths with temperatures as high as 55°C [50, 68] and a lack of withdrawal within 20 s has been frequently observed in 46°C water baths [24, 52, 101]. In the current study, the absence of heat response and capsaicin effect in 2 out 8 NHPs at 48°C water baths indicate that there may be a difference in the level of pain perception or subjective tolerance among these animals. Animal prescreening based on tail withdrawal responses might be valuable to select only responders to the noxious challenge for the subsequent imaging study, which may minimize inter-subject variability.

The large inter-subject variability may explain the marginal effect of capsaicin on tail withdrawal latency (see Fig 2B). Generally, capsaicin augments feelings of pain and reduces measures of pain threshold or increases subjective reports of pain [11, 20, 23–25]. The relatively limited effect in the animal studies here likely reflects inter-subject variability and the small number of animals used for the study. In addition, the effects of capsaicin on nociceptive processes are complex and involve combinations of increased nociceptive activity, hyperalgesia, allodynia and analgesia [12, 14]. In our study, tail withdrawal latency was recorded 15 min after subcutaneous injection of capsaicin into the tail. Fifteen minutes was chosen based on the study of Iadarola et al. and Butelman et al. [23, 88] with the expectation that direct nociceptive activity would reduce considerably after 15 min. That expectation, however, was not directly assessed and may not be applicable to subcutaneous delivery in nonhuman primates.

To compare the results obtained from the tail withdrawal behavioral assay and BOLD fMRI experiments within the same animals, it was found that one tail withdrawal non-responder (#3) consistently showed no potentiation effect in capsaicin-induced brain activation, whilst the other non-responder (#4) showed significant and robust increases in BOLD signal change following capsaicin application (see Fig 5). These interesting findings were attributed to the notion that a lack of reduction in tail withdrawal latency may subjectively reflect either the absence of pain sensation or the presence of higher tolerance to pain, but the latter may however still objectively render a positive response in an fMRI study. When animals organize behavioral responses to aversive stimuli under hypersensitization, the pain sensation is fairly complex involving multiple dimensional components (i.e. emotion, cognition). Any observed behavioral readouts measured by simply defined sensory modalities are likely a mere approximation to the overall pain responses [49]. Hence, in the assessment of novel analgesics complementary subjective and objective measurements might be necessary to better understand clinical outcomes.

It is noted that there are differences in experimental conditions between the tail withdrawal behavioral test and fMRI study and thus care needs to be exercised when comparing the results obtained from these two assays. Specifically, capsaicin was applied at two different anatomical location / route of administration (i.e. tail / subcutaneous versus forearm / topical), and the physiological condition of the animal was also varied (unanesthetized versus anesthetized). Stimulus-evoked tail withdrawal reflex response is a well-established behavioral assay where subcutaneous administration of capsaicin into the tail [65, 68] has found widespread application in recent studies for assessing efficacy of novel analgesic compounds [51, 66]. In addition, this behavioral model has improved our understanding of pain mechanism. As such, to bridge the current study with previous work, we also injected capsaicin subcutaneously in our tail withdrawal behavior tests. On the other hand, human translation of this NHP pain model being one important aspect of this fMRI study and thus, similar to the previous human fMRI study, capsaicin was administered topically at the forearm using patch formulation described by Mohr et al., [67]. Also, topical administration at the forearm was preferred over subcutaneous or intradermal injection to avoid direct systemic administration of capsaicin. Nonetheless, we believe that within-subject behavioral and fMRI data collected in this work has provided valuable complementary information on pain processing.

Technical constraints in the availability of animal cohort and imaging scanner restricted the sample size and also meant that three animals did not complete the vehicle testing arm of the study. A larger sample is likely to reveal more brain areas of significant activation. Thalamic activity, for example, was not observed in this study, which may be a direct consequence of lack of statistical power. The ROI analysis (see Fig 4) does indicate increased thalamic response following capsaicin application but that increase was, presumably, insufficient to reach threshold in the voxel-by-voxel analysis. In human studies, however, thalamic activation has been observed due to direct capsaicin-induced pain [88, 102], but was absent during capsaicin-induced allodynia to light brushing [88, 102–104]. Hence, the lack of thalamic activation observed in our study might also be reconciled with the occurrence of concomitant baseline activity associated with capsaicin-induced pain. However, the nature of thalamic activity in nonhuman primates is not yet fully understood and requires further investigation.

Despite the limitations, this capsaicin pain fMRI nonhuman primate model offers a number of potential benefits. First, it provides a means of driving pain matrix activation without delivering a directly tissue-damaging stimulus, which permits a refinement over noxious stimulus and better consistency with the 3Rs guiding animal research. Second, it affords a model of pain that reflects neuropathic mechanisms that are known to be involved in a variety of chronic pain disorders, and thus this model potentially provides a translational biomarker to evaluate the efficacy of novel analgesics. Nonetheless, it is important to acknowledge that the underlying neurobiology of chronic neuropathic pain is complex and thus additional studies are required to demonstrate the relevance of this acute model to study chronic pain. The BOLD responses and thermal hypersensitization observed in the current study were established within a very short experimental time frame, whilst chronic pain usually develops gradually over a period of days to weeks and lasts for years. Therefore, care must be exercised when translating our findings to chronic pain. The pathophysiology of chronic pain likely encompasses more complex and progressive changes, as the pain condition persists and adaptive processes in neural circuits are engaged to maintain homeostasis [105, 106]. For example, modulatory changes of physiology has been amply demonstrated for adult human motor cortex [107, 108], as well as somatosensory cortex in both normal subjects and patients with pathological pain [109]. Furthermore, within the clinical context, especially in patients with chronic pain, often many factors besides pathophysiology contribute to the clinical complaint of the dysfunction and thus influence the assessment of pain response; psychological, social, behavioral and cognitive aspects of the illness to name a few [39, 110–112].

Currently, there is much anticipation, and optimism, that new classes of analgesics, such as adenylyl cyclase inhibitor [113, 114], tetrahydrobiopterin inhibitor [115], selective sodium channel blocker [116, 117] and so forth, might have efficacy for chronic pain. Clinical investigation of these compounds, however, typically follows animal model testing for efficacy and safety reasons and there is, therefore, a need to develop appropriate animal models. Future studies might validate and enhance the current findings via the use of a wider spectrum of noxious stimuli and analgesic compounds that have limited, but known, efficacy against neuropathic pain. Examples might include antidepressant/antiepileptic class of drugs [34, 118]. With continuing success, this capsaicin pain fMRI nonhuman primate model may become a useful tool for early preclinical testing of novel compounds to relieve chronic neuropathic pains in patients.

## Supporting Information

S1 Fig Significant increases in BOLD responses to 42°C heat stimulus (post-capsaicin vs pre-capsaicin, p < 0.05, n = 5). Slices are shown in neurological orientation. Potentiation in BOLD responses is evident in the cerebellum, insula, primary and secondary somatosensory cortices, anterior cingulate cortex extending into medial frontal regions, mid-anterior cingulate cortex extending into posterior cingulate and prefrontal cortex. The observed brain activation pattern is highly comparable to those of seen in the group level analysis of full eight animals shown in Fig 3.
(TIF)

S2 Fig Significant increases in BOLD responses to 42°C heat stimulus (post-capsaicin compared to pre-capsaicin) vs (post-vehicle compared to pre-vehicle). The interaction effects are evident in the cerebellum, insula, primary and secondary somatosensory cortices, anterior cingulate cortex extending into medial frontal regions and mid-anterior cingulate cortex extending into posterior cingulate (p < 0.05, n = 5).
(TIF)

S1 File Full factorial analysis from the five animals completing all procedures. (DOCX)
