# Supplementary figures and images for: Distinct BOLD fMRI Responses of Capsaicin-Induced Thermal Sensation Reveal Pain-Related Brain Activation in Nonhuman Primates
Source: PLoS One. 2016 Jun 16;11(6):e0156805. doi: 10.1371/journal.pone.0156805 (PMC4911046; doi:10.1371/journal.pone.0156805)

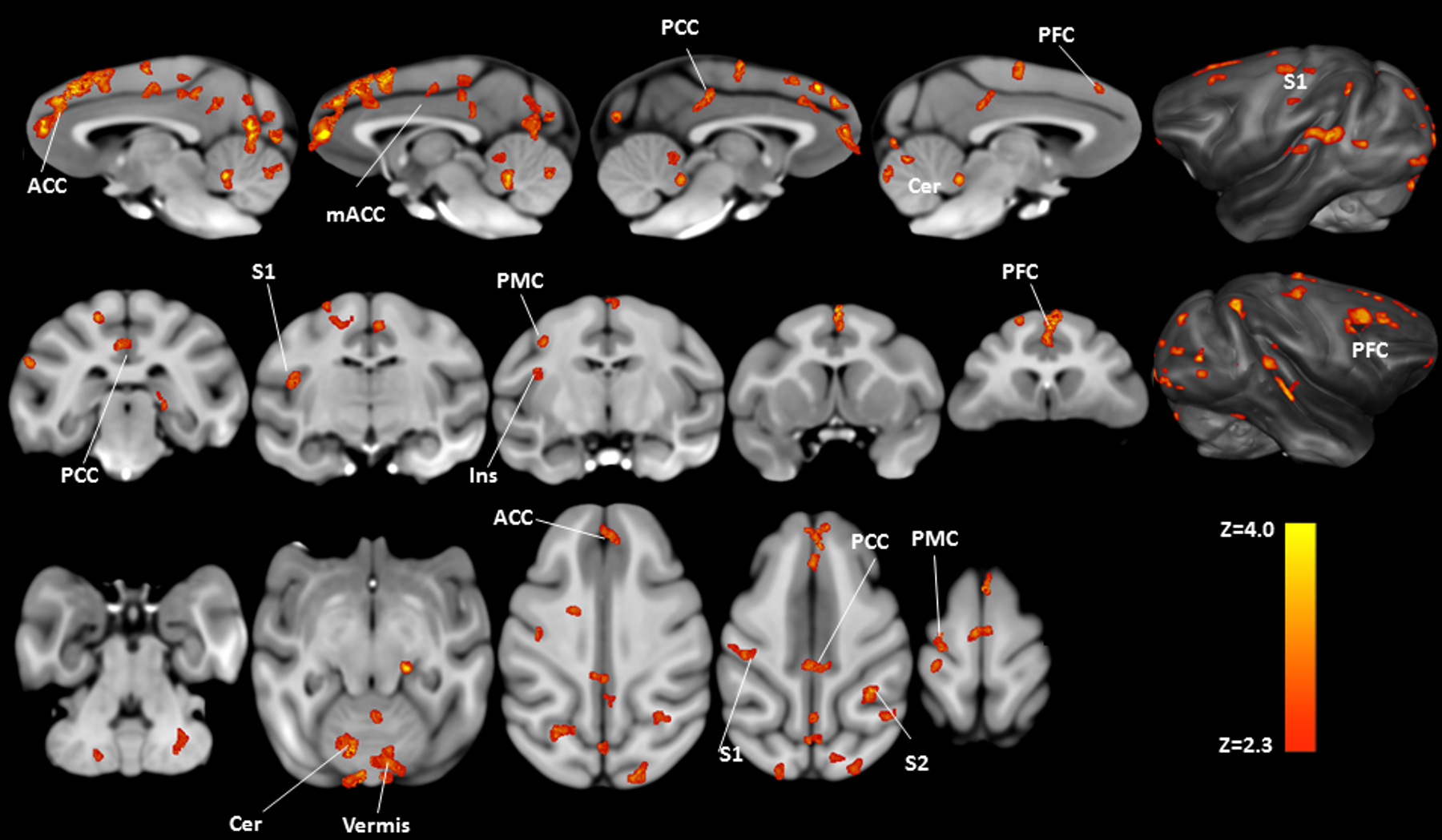

Supplement: S1 Fig — Slices are shown in neurological orientation. Potentiation in BOLD responses is evident in the cerebellum, insula, primary and secondary somatosensory cortices, anterior cingulate cortex extending into medial frontal regions, mid-anterior cingulate cortex extending into posterior cingulate and prefrontal cortex. The observed brain activation pattern is highly comparable to those of seen in the group level analysis of full eight animals shown in Fig 3. (TIF) [file pone.0156805.s001.tif]

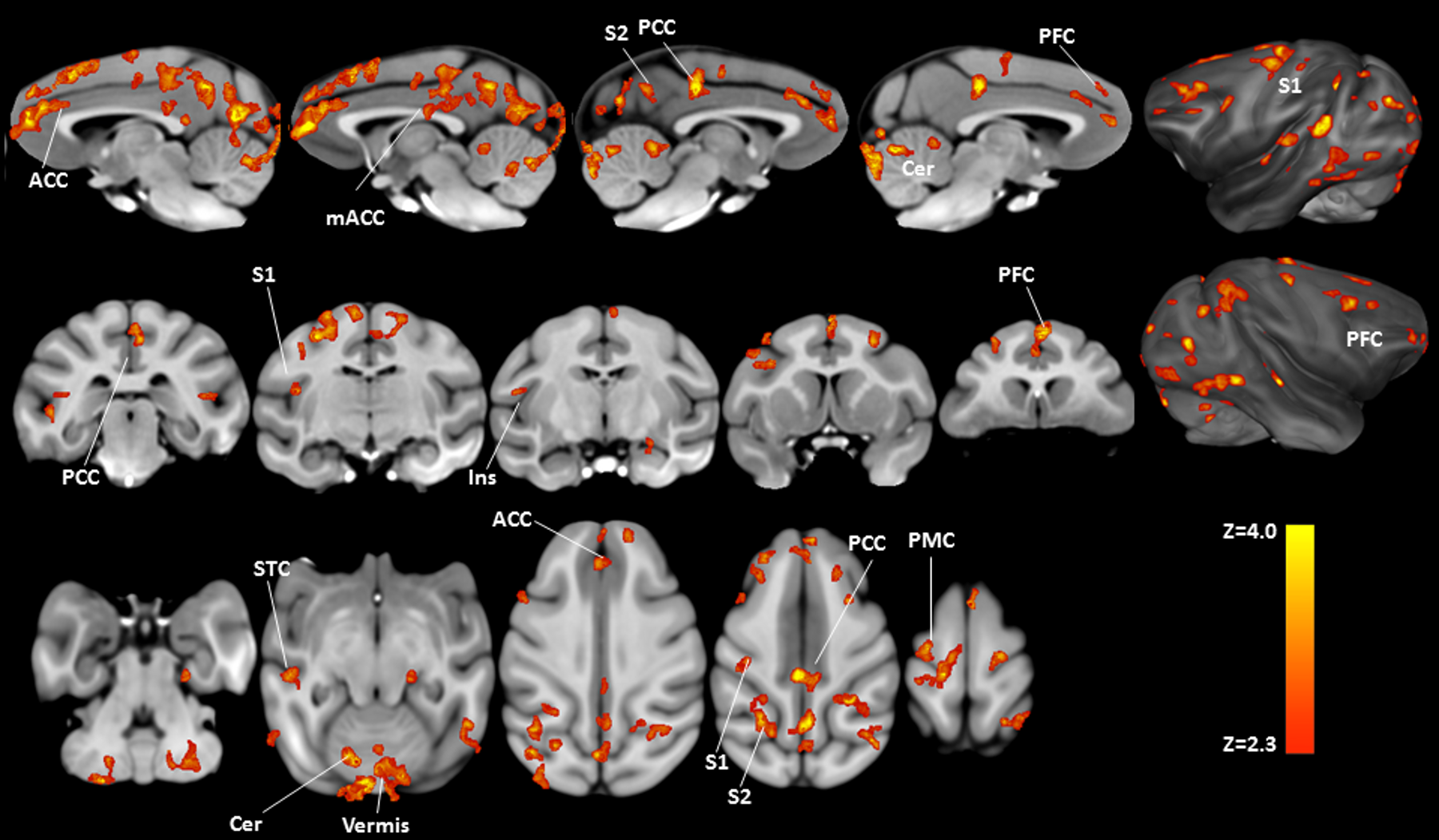

Supplement: S2 Fig — The interaction effects are evident in the cerebellum, insula, primary and secondary somatosensory cortices, anterior cingulate cortex extending into medial frontal regions and mid-anterior cingulate cortex extending into posterior cingulate (p < 0.05, n = 5). (TIF) [file pone.0156805.s002.tif]
