# Supplementary material for: Distinct BOLD fMRI Responses of Capsaicin-Induced Thermal Sensation Reveal Pain-Related Brain Activation in Nonhuman Primates
Source: PLoS One. 2016 Jun 16;11(6):e0156805. doi: 10.1371/journal.pone.0156805 (PMC4911046; doi:10.1371/journal.pone.0156805)
Supplement: S1 File — (DOCX) [file pone.0156805.s003.docx]

**Full factorial analysis from the five animals completing all procedures**

Data analysis performed in a balanced design using only the five animals that have completed all the repeated measure conditions.

**Methods:** A fully factorial repeated measure group comparison, including both the capsaicin and vehicle arms (n = 5 per arm), was conducted using a mixed-effect model (FSL FLAME 1+2) (<http://www.fmrib.ox.ac.uk/fsl>, [1]), in which the group mean of the differential BOLD responses to the 42 °C heat stimulus was determined (pre- and post-capsaicin application). Based on our previous approach [2], calculated group activation maps were thresholded at z > 2.0 with differences considered statistically significant at p < 0.05.

**Summary:** Brain regions with enhanced BOLD responses shown in both Figure S1 (main effect) and S2 (interaction effect) are associated with the known primary pain matrix. These findings further substantiate that capsaicin-induced hypersensitization is the primary driver of BOLD responses observed in this animal model.

**References:**

1. Smith SM, Jenkinson M, Woolrich MW, Beckmann CF, Behrens TE, Johansen-Berg H, et al. Advances in functional and structural MR image analysis and implementation as FSL. Neuroimage. 2004;23 Suppl 1:S208-19.

2. Mayhew SD, Hylands-White N, Porcaro C, Derbyshire SW, Bagshaw AP. Intrinsic variability in the human response to pain is assembled from multiple, dynamic brain processes. Neuroimage. 2013 Jul 15;75:68-78.
